# The Effects of Theta-Gamma Peak Stimulation on Sensorimotor Learning During Speech Production

**DOI:** 10.1162/NOL.a.22

**Published:** 2025-11-03

**Authors:** Birtan Demirel, Daniel Lametti, Noa Alony Gilboa, Charlotte J. Stagg, Kate E. Watkins

**Affiliations:** Wellcome Centre for Integrative Neuroimaging, Department of Experimental Psychology, University of Oxford, Oxford, UK; Medical Research Council Brain Network Dynamics Unit, University of Oxford, Oxford, UK; Department of Psychology, Acadia University, Wolfville, NS, Canada; Wellcome Centre for Integrative Neuroimaging, FMRIB, Nuffield Department of Clinical Neurosciences, University of Oxford, Oxford, UK

**Keywords:** adaptation, brain stimulation, sensorimotor learning, speech, transcranial alternating current stimulation (tACS)

## Abstract

Transcranial alternating current stimulation (tACS) is a noninvasive neuromodulatory tool that is thought to entrain intrinsic neural oscillations by supplying low electric currents over the scalp. Recent work has demonstrated the efficacy of theta-gamma phase-amplitude coupled tACS over primary motor cortex to enhance motor skill acquisition and motor recovery after stroke. Here, we wished to assess the efficacy of tACS delivered with 75-Hz gamma coupled to the peak of a 6-Hz theta envelope (theta-gamma peak; TGP) at an intensity of 2 mA peak-to-peak to enhance sensorimotor learning during speech production. Sensorimotor learning was measured by shifting the formant frequency of vowels in real-time as speech is produced and measuring the adaptation to this altered feedback. The study was a between-subjects, single-blind, sham-controlled design. We hypothesised that participants who performed the speech task while receiving TGP tACS over the speech motor cortex (*N* = 30) would show greater adaptation to altered auditory feedback than those receiving sham stimulation (*N* = 31). Contrary to this hypothesis, there was no effect of TGP tACS on adaptation to the upwards F1 shift in auditory feedback in either the final 30 trials of the learning phase or in the first 15 trials of the after-effect phase. However, a trend emerged in the TGP tACS group for greater retention of the adapted state and slower return to baseline F1 values in the after-effect phase. This finding was not predicted, and highlights the need for further investigation to deepen our understanding of the effects of TGP tACS on speech motor learning.

## INTRODUCTION

Noninvasive brain stimulation methods have provided promising results in enhancing the acquisition of novel motor skills and efficacy of rehabilitative interventions. Several studies demonstrated the effectiveness of transcranial direct current stimulation (tDCS) to enhance the rate and retention of learning (Reis et al., 2009). tDCS can improve upper limb use following stroke when used in conjunction with therapy (Allman et al., 2016). Similarly in the speech domain, it can aid speech therapy in post-stroke aphasia (Marangolo et al., 2013) and enhance fluency in people who stutter (Chesters et al., 2018; Moein et al., 2022). Recently, another form of brain stimulation, namely, transcranial alternating current stimulation (tACS), has also been shown to be effective (see Hu et al., 2022, for a systematic review and meta-analysis). Specifically, engaging dynamic activity linked to learning is a promising approach (Akkad et al., 2021; Rustamov et al., 2022), and indeed early data suggest that a specific form of tACS, gamma coupled to the peak of a theta envelope (theta-gamma peak; TGP), may increase learning with a larger effect size than seen in similar studies using tDCS (Akkad et al., 2021). To our knowledge, TGP tACS has not been used to improve sensorimotor learning in the speech motor domain. Therefore, the primary objective of the proposed study was to assess the efficacy of TGP tACS in enhancing sensorimotor learning in speech by using a sensorimotor adaptation task in young healthy controls. Positive evidence for the efficacy of TGP tACS in enhancing learning in the speech motor domain would suggest that it could be an effective form of stimulation when used in conjunction with speech fluency training in people who stutter and as support for other rehabilitation efforts that focus on speech production.

The task selected for this study involved sensorimotor learning in speech using an adaptation paradigm in which the auditory feedback of participants’ speech production is altered in real-time, leading participants to adjust their speech production in response to compensate for this change (Houde & Jordan, 1998; Lametti et al., 2018; Niziolek & Guenther, 2013; Tang et al., 2021). This form of adaptation investigates the processes that govern how we acquire speech motor patterns by integrating sensory feedback with production. Several previous studies employed speech or pitch adaptation tasks to investigate whether brain stimulation can enhance sensorimotor learning in fluent speakers (Deroche et al., 2017; Lametti et al., 2018; Li et al., 2023; Scott et al., 2020; Shum et al., 2011). Such tasks are a good choice for these investigations as it is possible to show gains in learning. In contrast, many other speech and language tasks show ceiling effects in young typically fluent speakers, which limits the possibility of showing positive effects of brain stimulation (e.g., Wiltshire & Watkins, 2020).

Speech adaptation tasks alter feedback by shifting the formant frequencies that characterise vowel identity during the steady-state portion of syllable production (Houde & Jordan, 1998). The acoustic spectrum of a speech syllable contains several formants representing acoustic energy around a particular frequency corresponding to specific resonances in the vocal tract. Vowels are characterised by the first two formants (F1 and F2). When the frequency of the formants is shifted, the speaker compensates by altering speech production (Purcell & Munhall, 2006). For example, when speakers utter a word such as “head” and the frequency of F1 is decreased or increased, they may perceive their production as sounding closer to the vowel sounds contained in words like “had” or “hid.” To achieve production closer to the intended vowel, the speaker compensates by adjusting the formant frequencies in their speech production in the direction opposite to the shift applied to feedback. Speakers are rarely aware of the frequency shift applied to the feedback, and their adaptive speech responses are not affected by explicit cognitive strategies (Munhall et al., 2009). The neural correlates of speech motor adaptation involved in executing articulatory commands, predicting their sensory consequences, and integrating sensory feedback for adapting subsequent movements likely involves ventral premotor and primary motor cortex, the cerebellum, and inferior parietal and superior temporal regions (Parrell et al., 2017; Segawa et al., 2015; Tourville et al., 2008).

Recently, the speech adaptation task has been used in conjunction with noninvasive brain stimulation to investigate the causal contribution of these brain regions to this form of sensorimotor learning (Lametti et al., 2018; Scott et al., 2020; Shum et al., 2011; Tang et al., 2021). One study used anodal tDCS to increase cortical excitability of the representations of the speech articulators in left primary motor cortex (M1) and of the right posterior lobe of the cerebellum during speech motor adaptation (Lametti et al., 2018). During speech production, participants heard their feedback in real-time with an increase in F1 frequency. Relative to a group who did not receive any stimulation (sham control), the group who received anodal tDCS over the cerebellum compensated for the altered feedback by decreasing the frequency of F1 to a greater extent; they did not change the frequency of F2. In contrast, the group who received anodal tDCS over M1 compensated for the altered feedback by decreasing F1 and increasing F2 to a significantly greater extent than the sham control group. These results were interpreted as indications of distinct roles for the cerebellum and M1 in sensorimotor learning. Specifically, it was suggested that the cerebellum involves compensating for the acoustical error (shift in F1 frequency) alone, and M1 generates more global adaptation in response to altered auditory feedback by moving production towards that of a target vowel rather than simply offsetting the acoustical error in the feedback (Lametti et al., 2018).

The current study expands on this previous work by exploring the efficacy of another kind of noninvasive brain stimulation, namely, tACS, to facilitate the sensorimotor learning of speech. tACS supplies low currents across the brain that result in weak modulation of membrane voltage in a temporally coordinated manner (for a review, see Antal & Paulus, 2013). The sinusoidal currents delivered during tACS are thought to mimic and perhaps promote intrinsic neural oscillations when delivered in a physiologically relevant manner. This entrainment of neural oscillations can provide efficient communication between neural networks so that presynaptic inputs are synchronised to arrive during moments of low inhibition thereby promoting the likelihood of action potentials occurring in the postsynaptic network (Fries, 2005, 2015). This modulation is analogous to pushing a swinging pendulum at the right time to be able to intervene more efficiently and may be adequate to entrain neurons at the tACS frequency. Previous work showed that tACS improved motor skill learning, increased the magnitude of vocal compensation, reduced symptoms of Parkinson’s disease, and improved phonological processing and reading accuracy in dyslexia (Akkad et al., 2021; Guerra, Asci, et al., 2020; Marchesotti et al., 2020; Schilberg et al., 2018).

The efficacy of tACS is thought to be enhanced by coupling the frequency of the stimulating current with the frequency of intrinsic neuronal oscillations. In hippocampal-dependent skill learning, gamma activity at 75 Hz coupled to the peak of slower, theta activity (6 Hz) is common (Bragin et al., 1995; Colgin, 2015; Lasztóczi & Klausberger, 2014). For instance, Lopes-dos-Santos and colleagues (2018) found that during memory encoding, 60–80 Hz activity, which increases significantly, is coupled to the peak of the underlying theta oscillation. This suggests that the precise relationship between gamma activity and theta phase may be crucial for its function. In M1, gamma activity, particularly that at approximately 75 Hz, is prokinetic, being increased during actual rather than imagined movement (see Nowak et al., 2018, for a review) and substantially enhanced in patients with dyskinesias (Swann et al., 2018). Similar to hippocampal-dependent learning, gamma coupled to the peak of a theta envelope appears to be important in M1-dependent learning: tACS consisting of a 75-Hz sinusoid present during the peak of a 6-Hz theta envelope delivered over M1 was demonstrated to increase motor skill acquisition in a thumb abduction task in healthy adults compared with sham and gamma coupled to the trough of a theta envelope (Akkad et al., 2021). Furthermore, motor recovery after stroke was recently linked to an increase in M1 theta-gamma phase-amplitude coupling (Rustamov et al., 2022).

In the current study, we hypothesised that people who perform the speech task with altered auditory feedback while receiving TGP stimulation over the speech motor cortex in left M1 would show greater compensatory responses to altered auditory feedback compared with those receiving sham tACS. We increased the frequency of the F1 feedback and investigated the magnitude of expected decreases in F1 and increases in F2 produced in response. The directions of these changes in response to increased F1 frequency feedback was based on those described in previous work (Lametti et al., 2018; MacDonald et al., 2011; Tang et al., 2021).

## MATERIALS AND METHODS

### Participants

We recruited healthy people who were 18 to 50 years old, able to give informed consent, and grew up speaking English. We excluded people with impaired hearing or a history or current diagnosis of dyslexia or other language or speech disorder, because weaker magnitudes of compensatory responses to altered auditory feedback have been shown in children with dyslexia (van den Bunt et al., 2018) and in a group of people who stutter (Cai et al., 2012).

This research expands on a previously reported finding that tDCS improves sensorimotor learning in speech following an increase in F1 feedback (Lametti et al., 2018). In this previous study, 20 participants received 2-mA anodal tDCS over the ventral motor cortex corresponding to the representations of speech articulators and 20 received sham tDCS. Participants who received active tDCS significantly reduced their F1 and increased their F2 production during the learning phase to a greater extent than the reductions seen in the group who received sham stimulation. An effect size (Cohen’s *d*) of 0.65 was reported for the group difference in F1 reduction, averaged across the learning and after-effect stages of the experiment (note the after-effect stage involves removal of the altered feedback and did not show a significant group difference, so this may be a conservative effect size). For the current study, a power calculation (G * power 3.1) indicated that 30 participants in each group should be sufficient to detect the same sized group difference in reduction in F1 in response to an upwards shift in F1 feedback, significant at 5% (one-tailed) with 80% power. It is worth noting that compared with sham stimulation, TGP tACS over the hand representation in M1 (Akkad et al., 2021) during a thumb abduction task resulted in 26% greater acceleration gain from baseline in motor learning, which was a large effect (Cohen’s *d* = 0.98).

### Procedure

The study used a between-subjects, single-blind, sham-controlled design. Participants engaged in a speech production task requiring them to read aloud words presented on a computer screen. Speech was recorded via a head-mounted microphone (Shure WH20) placed 4–7 cm away from the right corner of the participant’s mouth. Each word was presented for 1,500 ms on the computer screen independently, and the intertrial interval was set at 750 ms. Words appeared in a pseudorandom order, an equal number of times. After each utterance, participants heard their voice over headphones (Sennheiser HD 380 pro) with an imperceptible delay (27 ms; Kim et al., 2020). MATLAB Mex-based program Audapter (with MOTU Microbook IIc external soundcard) was used to alter the auditory feedback of speech equal to 110 mel (Tang et al., 2021). We predicted that when the altered auditory feedback was applied, participants would compensate for the mismatch between the expected auditory target and altered auditory feedback by reducing the F1 frequency. Based on previous work, we also expected participants to increase the F2 frequency of their speech production (Lametti et al., 2018; Tang et al., 2021). This adaptation is also typically present in the initial few trials during the after-effect phase when auditory feedback returns to normal.

**Figure 1.**
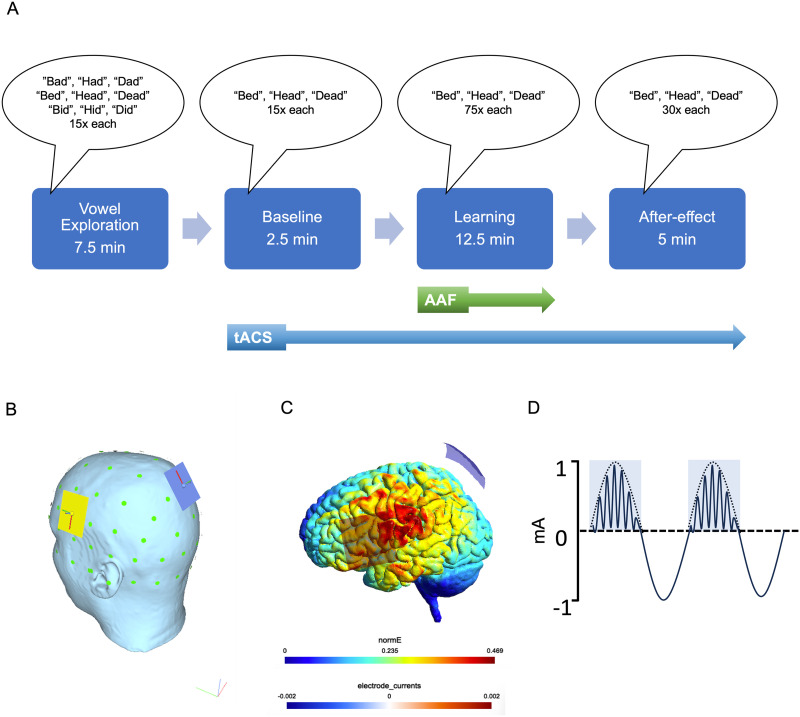
Experimental procedure. (A) Schema depicts the experimental design with four phases: vowel exploration, baseline, learning, and after-effect. Participants read one-syllable words from a computer screen. tACS was delivered during the baseline, learning, and after-effect phases. The total time of the experiment lasted approximately 1 hr. (B) One electrode (5 × 5 cm) was centred over the ventral motor cortex (yellow, approximately FC5; see text for details on positioning) to target the speech articulators. The other electrode was centred over the posterior midline (blue, Pz). (C) Direct current flow simulation: estimated distribution of the current flow acquired at current peaks. Norm_E bar represents the strength of the electric field. Electrode_currents bar depicts the amplitude of the electric current, 2 mA for the anode electrode; −2 mA for the cathode electrode. (D) tACS waveform: gamma frequency stimulation was delivered during the peak of a theta envelope. tACS = transcranial alternating current stimulation; AAF = altered auditory feedback.

The stimulating electrodes were attached to the head before the start of the experiment. The experiment included four phases (depicted in Figure 1A): vowel exploration, baseline, learning, and after-effect. The first phase allowed participants to explore vowel space when they produced “dead,” “bed,” “head,” “dad,” “bad,” “had,” “did,” “bid,” “hid” 15 times for each with normal feedback. The rationale of this phase was to record the formant frequencies used for F1 and F2 productions of these previously learned words for each participant, which then allowed us to compare the possible frequency adjustments towards these words in response to altered auditory feedback. After the vowel exploration phase, the stimulator was turned on and there was a short break during which we confirmed that the participant was comfortable and ready to proceed. The baseline phase started and participants produced the words “head,” “bed,” and “dead” 15 times each, containing the same vowel /ε/, with normal feedback, which gave a measure of production with stimulation before learning started. In the learning phase, participants produced “head,” “bed,” and “dead” 75 times each with altered auditory feedback. Finally, participants produced “head,” “bed,” and “dead” 30 times each again with normal feedback in the after-effect phase, which gave a measure of the maintenance of sensorimotor learning in speech. There were 30-s breaks after every 45 utterances. tACS was delivered for approximately 20 mins, starting during the baseline phase and continuing during the learning and after-effect phases.

### tACS

The somatotopy of the representations of the speech articulators along the precentral gyrus is such that a dorsal laryngeal representation is found ventral to the hand representation, followed by representations of the lip and tongue moving ventrally and anteriorly along the gyrus, with a ventral laryngeal representation located on the subcentral gyrus (Eichert et al., 2020). We delivered stimulation over the ventral two-thirds of the precentral gyrus to target these speech articulators as shown in Figure 1B. One electrode (5 × 5 cm) was placed on the left side of the head, with the top right (posterior) corner of the electrode positioned at a point one-third of the length along a line connecting the vertex to the tragus (Lametti et al., 2018) and the other electrode was placed over the posterior midline (Pz). With this montage setup, the distribution of the current flow was simulated using SimNIBS software which is shown in the Figure 1C. We have confidence that the motor representations of the speech articulators are encompassed by this large electrode, regardless of the size of the head. Therefore, the electrode’s size is advantageous in addressing the variation between individuals in the precise locations of these representations, as it ensures that they are adequately covered.

Participants were randomised to receive either tACS or sham stimulation and these assignments were concealed from participants. A researcher not involved in data collection generated a randomisation code for participants using a block size of four to ensure a close balance of participants allocated to each stimulation group throughout the study. Groups were balanced for gender. Our intention was that assignment of participants to receive tACS or sham stimulation was concealed from researchers involved in delivering the stimulation and analysing the data (i.e., that the study was double-blind). The visibility on the computer monitor of the theta-gamma waveform meant that this was not possible for 22 participants, in whom we consider data collection to have been conducted in a single-blind manner. This is a deviation from our original preregistration protocol. However, the researcher was blinded to the group allocation during data analysis for all participants, as planned.

The active stimulation condition included gamma frequency stimulation at 75 Hz delivered during the peak of a 6-Hz theta envelope at an intensity of 2 mA peak-to-peak as depicted in Figure 1D (Akkad et al., 2021). Unlike the active stimulation condition in which the entire theta-gamma peak amplitude coupled waveform was delivered, the sham stimulation condition included the delivery of only the beginning of the waveform, specifically 10 s of continuous sinusoidal 6-Hz stimulation. In this way, participants felt only the initial sensation of stimulation at the start of the waveform, which is a similar sensation to the active condition without causing any further neural modulation. Therefore, this protocol was thought to be sufficient to conceal the stimulation condition from the participant. Once the session ended, the participants completed a treatment identification questionnaire to assess the effectiveness of the stimulation condition concealment protocol as well as a questionnaire on possible side effects. We intended for the experimenter to complete the same treatment identification questionnaire, but as it was not possible for the experimenter to be blind during data collection in all participants, this was not done.

### Analysis of Data

MATLAB and the software package Praat (Boersma & Weenink, 2001) were used to analyse speech acoustics. Speech was recorded at 16000 Hz using custom MATLAB scripts. The centre of each vowel was detected through a visual examination of the speech waveform in MATLAB. Finally, we used Praat to perform linear predictive coding (LPC) to measure each vowel’s average F1 and F2 values over a 25 ms (400 samples) segment at the centre of each vowel. LPC orders were chosen per subject to minimise variability in the extracted formants. We removed data points that were errors (wrong word repeated, coughs, no or incomplete responses) and where the estimated formant frequencies exceeded 3 *SD*s of the mean for that phase of the experiment for each individual. All data were checked for normality using the Shapiro-Wilk test. We planned to transform data that was not normally distributed.

Our statistical analysis plan was based on that of Lametti et al. (2018). We first demonstrated that our two groups produced acoustically similar speech at baseline. The mean baseline frequencies for F1 and F2 (in Mel) were compared between the two groups using *t* tests (two-tailed, uncorrected to avoid a false negative). We did not expect a significant difference between groups in either measure. In case of a difference in measures at the onset, any individuals who exhibit outlier frequencies in F1 or F2 at baseline (defined as >3 *SD*s from the mean) would be substituted, before the unblinding.

Sensorimotor learning for each participant was calculated by the mean change from baseline in F1 and F2 frequencies separately at the end of the learning phase (last 30 trials) and at the start of the after-effect phase (first 15 trials). These changes (learning) were assessed within each group separately using directional one-tailed *t* tests against no change (i.e., zero; reduced F1 and increased F2 frequencies were expected, *p* < 0.05).

Our main hypothesis predicted a significantly greater magnitude of adaptation in F1 in the TGP tACS group relative to the sham group as previously seen using tDCS over M1. This was tested separately for the end of learning and initial after-effect trials with directional *t* tests (*p* < 0.05). It is worth noting that the previous study (Lametti et al., 2018) found a significant group difference only for the trials at the end of learning, and the difference in the initial after-effect trials was not significant though the pattern of results was similar.

We also tested for group differences in the magnitude of adaptation in F2 in the end of learning and initial after-effect trials, respectively. We used nondirectional *t* tests (*p* < 0.05) since there was no significant difference between the groups receiving tDCS over M1 and the sham group in the magnitude of adaptation in F2 in the previous study at either phase of the experiment (Lametti et al., 2018).

Previous work showed that participants who experienced an increase in F1 frequency during feedback produced combined changes in F1 and F2 that resulted in moving speech production closer to a target vowel in vowel space (Lametti et al., 2018; Tang et al., 2021). We also tested whether the magnitude of this combined change differed between groups in the current study. For each individual, we calculated the Euclidean distance in F1–F2 vowel space between the productions of “head,” “bed,” and “dead” during the last 30 trials of the learning phase and the first 15 trials of the after-effect phase and productions of “hid,” “bid,” and “did” during the vowel exploration phase (Lametti et al., 2018). The magnitude of the combined F1–F2 change was compared between groups using a nondirectional *t* test (*p* < 0.05) as there was no difference between the M1-tDCS and sham groups in the previous study in the learning and after-effect phases of the study.

## RESULTS

Data were obtained in 62 participants (32 in sham group). Data from one participant was excluded from the sham group due to stuttering during the task, and the participant subsequently reported a history of childhood stuttering. Due to a technical error, data from the after-effect block were lost for another participant in the TGP tACS group. The TGP tACS group therefore comprised 30 participants (15 women) with data from 29 available for the after-effect phase, and the sham group comprised 31 participants (16 women). The groups were also matched for age (Table 1).

**Table 1.** Group averages for age and frequencies

**Sex**	**15 women**	**16 women**
Age (yr)	23.4 ± 4.9	23.7 ± 6.7
Baseline F1	747 ± 86.8	767 ± 73
Baseline F2	1468 ± 105.5	1427 ± 81.6
End of learning F1^a^	713 ± 81.3	736 ± 71.7
End of learning F2^a^	1480 ± 100.7	1433 ± 85.2
Start of after-effect F1^b^	720 ± 81.3	750 ± 68.8
Start of after-effect F2^b^	1478 ± 103.5	1435 ± 82.8

*Note*. Means ± *SD* are reported. Frequencies are reported in Mel.

^a^ Last 30 trials of learning phase (trials 241–270).

^b^ First 15 trials of after-effect block (trials 271–285).

Prior to analysis, formant values exceeding 3 *SD*s from the mean F1 or F2 value for each participant in each block were removed (∼0.02% of the data) to exclude trial-level anomalies. Please note, a separate group-level outlier (reported in the exploratory analysis) refers to a participant’s mean adaptation score that was more than 3 *SD*s from the group mean. To verify group comparability at baseline, we compared the F1 and F2 formant frequencies during the baseline phase. The groups produced acoustically similar speech at baseline for F1 and F2 frequency (F1: *t*(59) = −0.96, *p* = 0.34; two-tailed, uncorrected; F2: *t*(59) = 1.70, *p* = 0.09; see Table 1).

### Changes in F1 Production

**Figure 2.**
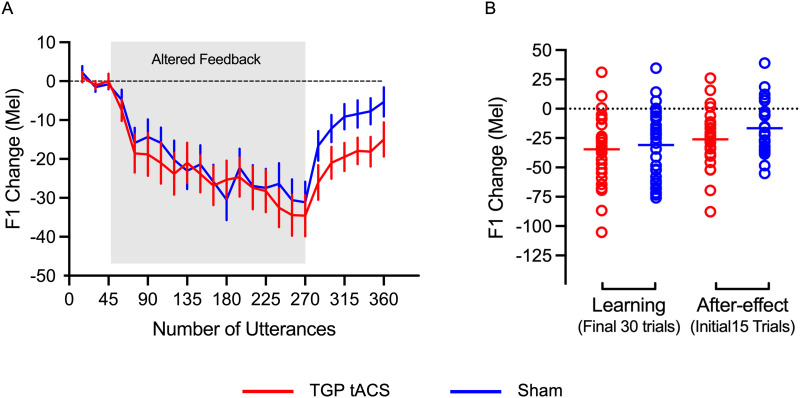
Changes in production of F1 due to sensorimotor learning. (A) Changes in F1 production in response to increased F1 feedback are shown for baseline, learning, and after-effect phases. (Note altered feedback was applied only during the learning phase, shaded area.) Each data point represents the mean F1 frequency for 15 utterances with error bars indicating the standard error of the mean. A dashed line indicates the mean of the baseline trials. (B) The average changes in F1 frequency for the final 30 trials of the learning phase (trials 241–270) and the initial 15 trials of the after-effect phase (trials 271–285). Solid lines show the means for each group, the open circles indicate individual participant data. Red indicates TGP tACS group data and blue data from the sham group. TGP tACS = theta-gamma-peak transcranial alternating current stimulation.

Figure 2A shows the change in produced F1 frequency during the learning phase (trials 46–270; shaded region) and the after-effect phase (trials 271–360), compared with the baseline F1 values (trials 1–45). As expected, by the end of the learning phase, both groups showed a significant decrease in F1 frequency from baseline in response to an upwards shift in F1 feedback (one-sample *t* test against zero, TGP tACS group *t*(29) = −6.65, *p* < 0.001, Cohen’s *d* = −1.21, one-tailed; sham group *t*(30) = −5.90, *p* < 0.001, Cohen’s *d* = −1.06, one-tailed; Table 1 and Figure 2B). The magnitude of average adaptation in the TGP tACS group was 31% relative to the 110 Mel shift (Observed Shift / Total Induced Shift × 100), and for the sham group was 27%. This adaptive response in F1 was still evident in the first 15 utterances in the after-effect phase in both groups (TGP tACS group *t*(28) = −5.82, *p* < 0.001, Cohen’s *d* = −1.08; sham group *t*(30) = −4.37, *p* < 0.001, Cohen’s *d* = −0.78; see Table 1 and Figure 2B).

To test our hypothesis that TGP tACS would lead to significantly greater adaptation in F1 than sham stimulation, the means for the two groups at the end of the learning phase and the beginning of the after-effect phase were compared using directional two-sample *t* tests. The amount of adaptation in each group did not differ for either phase (end of learning: directional *t*(59) = −0.5, *p* = 0.31, Cohen’s *d* = −0.13; start of after-effect: *t*(58) = −1.61, *p* = 0.06, Cohen’s *d* = −0.42). In sum, TGP tACS had no significant effect on the magnitude of F1 adaptive changes in response to an F1 feedback perturbation.

### Changes in F2 Production

**Figure 3.**
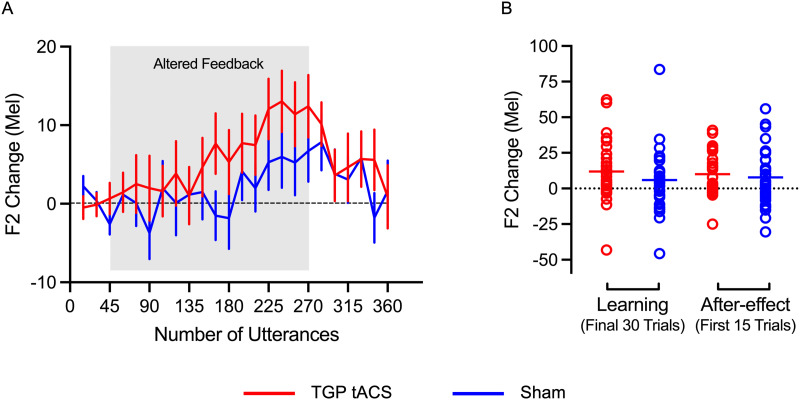
Changes in F2 production due to sensorimotor learning. (A) Changes in F2 frequency during production throughout the baseline, learning, and after-effect phases. (B) Average changes in F2 across the last 30 trials of the learning phase (trials 241–270) and initial 15 trials of the after-effect phase (trials 271–285). Refer to Figure 2 caption for further information.

Previous studies reported an increase in F2 frequency during vowel production in response to an upwards shift in F1 in the auditory feedback (Lametti et al., 2018); the magnitude of this shift is typically smaller and less consistently observed among individuals and studies (Lametti et al., 2018; Tang et al., 2021). Based on previous work (Lametti et al., 2018), we did not expect TGP tACS over speech motor cortex to modulate the size of the adaptation in F2 relative to sham. Figure 3A shows the change in produced F2 frequency from baseline, through the learning when the F1 upwards shift was applied to feedback and during the after-effect phase when normal feedback was restored.

Figure 3B shows the mean changes in F2 from baseline for each group. A Shapiro-Wilk test indicated that the data for the magnitude of the F2 shift at the end of the learning phase significantly deviated from a normal distribution in both the TGP tACS group (W = 0.92, *p* = 0.04) and the sham group (W = 0.88, *p* < 0.01). For the after-effect phase, only the data in the TGP tACS group was not normal (TGP tACS group: W = 0.92, *p* = 0.045; Sham tACS group: W = 0.97, *p* = 0.68). Additionally, an outlier was identified in the sham group with a value of 83.59 (Figure 3B), which is more than 3 *SD*s above the group mean (*M* = 5.94, *SD* = 21.39). Given that normality violations were not uniform across all conditions, we did not transform the data as originally planned and instead employed the Wilcoxon signed-rank test to compare changes against baseline and the Mann-Whitney U test to compare groups, because these nonparametric tests do not assume normality, are more robust to deviations and outliers, and thus provide a more interpretable solution in this case.

A Wilcoxon signed-rank test showed a significant change in F2 from baseline for the TGP tACS group (V = 373, *p* < 0.001, *r* = 0.71) and the sham group (V = 334, *p* = 0.047, *r* = 0.35), with a numerically larger effect in the TGP tACS group. To compare the adaptation between groups, Mann-Whitney U tests indicated no differences in the magnitude of adaptation in F2 either at the end of the learning phase or in the initial after-effect trials (end of learning: U = 549, *p* = 0.23, Cohen’s *d* = 0.18; start of after-effect: U = 504, *p* = 0.43, Cohen’s *d* = 0.12). In sum, both groups showed a significant increase in F2 value from baseline during learning, but there were no significant differences between the groups during the learning and after-effect phases.

### Changes in F1–F2 Vowel Space

**Figure 4.**
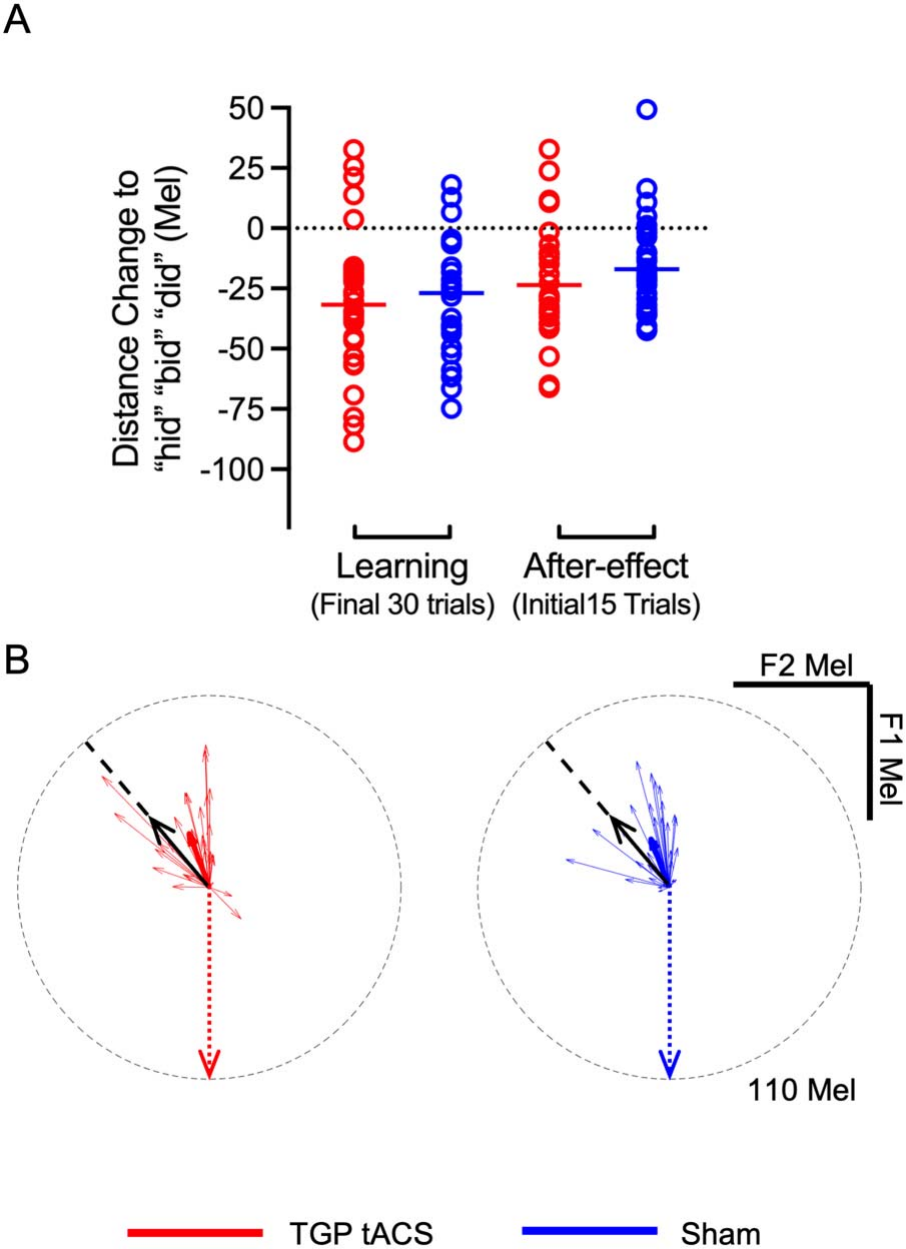
Distance change in F1 and F2 towards “hid,” “bid,” and “did” in vowel space in response to an upwards F1 shift in feedback. (A) The change in the Euclidean distance (measured in Mel within the F1–F2 space) between the baseline production of “head,” “bed,” and “dead” and the altered production, which shows that participants move their production closer to “hid,” “bid,” and “did.” See Figure 2 for further information. (B) Red and blue vectors illustrate the compensatory adjustments in vowel production for TGP tACS and sham groups in the learning phase. Individual data are represented by thin vectors, while the thick vectors show the group average. The directions of the individual vectors were determined by their change in the participant’s F1–F2 vowel space, and their magnitudes were adjusted by values in their normalised F1–F2 change. The black arrow indicates the required change direction from the vowel in the word “head” towards the vowel in “hid,” and the dotted arrow shows the direction and magnitude of the formant shift (110 Mel) applied to feedback during learning. A light grey circle marks the 110 Mel extent of the applied shift, giving a reference to the size of the participants’ adjustments.

In previous studies, the combined adaptive changes in F1 and F2 in response to altered F1 feedback resulted in moving speech production closer to the production of an existing vowel in vowel space but stimulation over the left speech motor cortex did not affect this response relative to sham stimulation (Lametti et al., 2018; Tang et al., 2021). To assess this in the current study, we calculated the Euclidean distance in F1–F2 vowel space between the productions of “head,” “bed,” and “dead” during the last 30 trials of the learning phase and the first 15 trials of the after-effect phase and productions of “hid,” “bid,” “did,” during the vowel exploration phase. The change in the Euclidean distance for productions of “head,” “bed,” “dead,” towards “hid,” “bid,” “did” in F1–F2 space during the learning phase is visualised in Figure 4A while Figure 4B shows the magnitude and direction of production in F1–F2 vowel space. The magnitude of the combined F1–F2 change was compared between groups using a nondirectional *t* test. Each group reduced the distance between the two vowels in vowel space but the groups did not differ in terms of the magnitude of these changes in either the learning phase (*t*(59) = −0.63, *p* = 0.53, Cohen’s *d* = −0.16) or the after-effect phase (*t*(58) = −1.14, *p* = 0.25, Cohen’s *d* = −0.29). Thus, TGP tACS had no effect on the magnitude or the direction of combined changes in F1 and F2 in response to the altered feedback.

### Exploratory Analysis on the After-Effect Phase for F1

Inspection of Figure 2A reveals a difference between the groups during the after-effect phase; compared with the sham group, the adaptive response in the TGP tACS group was slower to return to baseline. In an exploratory analysis, we compared the mean change in F1 frequency during this phase between groups (see Figure 3). Shapiro-Wilk test for normality confirmed these data were normally distributed for both TGP tACS (W = 0.97, *p* = 0.660) and sham groups (W = 0.96, *p* = 0.25). A two-tailed *t* test revealed a statistically significant difference between the two groups for the after-effect phase (*t*(58) = −2.02, *p* = 0.048, Cohen’s *d* = −0.52).

**Figure 5.**
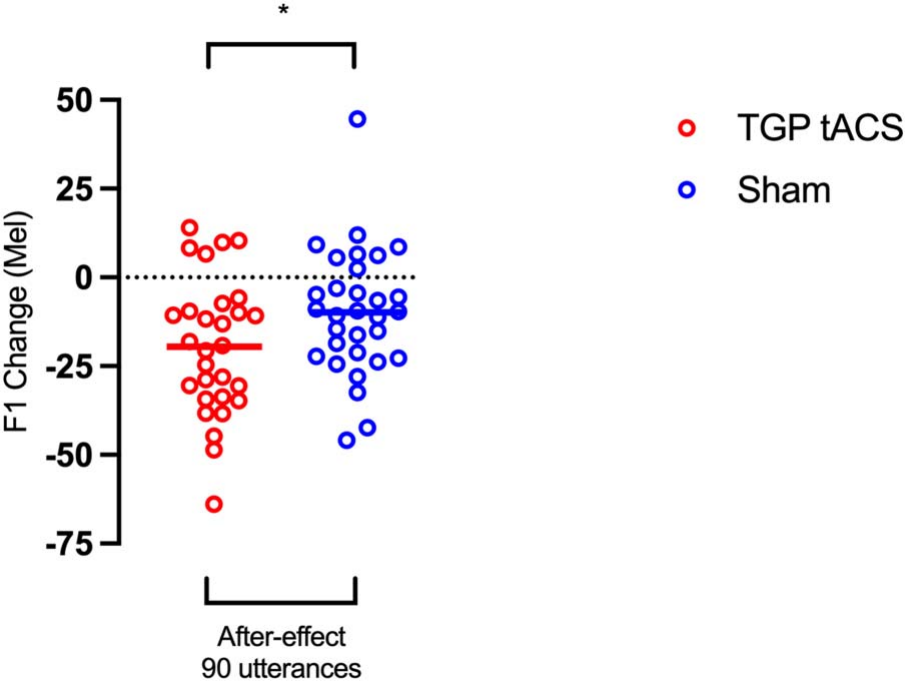
Group differences in the after-effect phase. F1 mean changes in all 90 utterances of the after-effect phase (trials 271–360) for both groups, TGP tACS and sham. The statistically significant difference between the groups is marked with an asterisk (*p* < 0.05). The colour coding remains the same as that outlined in Figure 2.

Examination of Figure 5 revealed an outlier in the sham group, with a maximum value of 44.63, which is more than 3 *SD*s from the group mean (43.24; *M* = −9.89, *SD* = 17.71). When we excluded the outlier the group difference was no longer significant (*t*(57) = −1.76, *p* = 0.084, Cohen’s *d* = −0.46).

### Exploratory Analysis of Motor Variability

To evaluate whether TGP tACS modulated trial-to-trial motor variability, we analysed the coefficient of variation (CV = 100 * *SD* / mean) of F1 frequency (Tang et al., 2022). We used all 45 trials from baseline, 45 from the end of adaptation, and 45 from the beginning of the after-effect phases since *SD* can only be compared for similar sample sizes. CVs were then entered into repeated-measures analyses of variance with phase as a within-subject factor and stimulation group (TGP tACS, sham) as a between-subject factor.

For F1, there was a significant interaction between Phase and Group (*F*(2, 116) = 4.82, *p* = 0.01), due to higher CV in the TGP tACS group for the adaptation (*M* = 4.18%, *SE* = 0.29) and after-effect (*M* = 3.98%, *SE* = 0.30) phases relative to baseline (*M* = 3.34%, *SE* = 0.24; planned pairwise comparisons: baseline vs. adaptation, *p* < 0.001; baseline vs. after-effect, *p* = 0.002; adaptation vs. after-effect, *p* = 0.91). There were no significant changes in CV among the different phases in the sham group (baseline *M* = 3.14%, *SE* = 0.23; adaptation *M* = 3.26%, *SE* = 0.29; after-effect *M* = 3.13%, *SE* = 0.29). Phase was a significant main effect (F1: *F*(2, 116) = 7.24, *p* = 0.001), but group was not (*p* = 0.08).

These findings suggest that TGP tACS increased variability in F1 during the altered feedback adaptation phase and that this effect persisted into the start of the washout phase when normal feedback was restored. Motor variability is often considered to reflect noise in the system and to be detrimental to movement performance. Conversely, motor variability can facilitate motor learning (Wu et al., 2014). Either way, the increase in motor variability in the TGP tACS group did not translate into enhanced sensorimotor learning on this task relative to the group who received sham and showed little change in motor variability. Nevertheless, it would be interesting to pursue this finding in future work and with other forms of noninvasive brain stimulation.

### Blinding and Adverse Effects

Our original intention to run a double-blinded study was not feasible since the waveform of the tACS was visible to the researcher interacting with the participant in at least 22 datasets (see Materials and Methods). The group allocation was concealed from the researcher during analysis of all speech files. To verify whether group allocation was successfully concealed from participants, we carried out a chi-squared test on the association between group assignment and correct responses to the stimulation condition. This indicated successful blinding (chi-squared = 2.08, *p* = 0.15), with 15 out of 31 participants in the sham group and 20 out of 30 participants in the TGP tACS group correctly reporting the stimulation protocol. In support of this, no significant group differences were found in any of the reported adverse effects between the groups (*p* > 0.05, uncorrected). This suggests that the stimulation protocol did not systematically influence any perceived adverse effects. It is worth noting that most participants reported no adverse effects and those that did rated them as very mild with the most common being mild tingling.

## DISCUSSION

In the present study, we evaluated the efficacy of a novel brain stimulation protocol, tACS with a 75-Hz gamma frequency coupled to the peak of a 6-Hz theta envelope, on sensorimotor learning in speech. We administered TGP tACS over the articulatory representation in the left sensorimotor cortex to investigate whether this would enhance the expected adaptation response to an upwards shift in F1 frequency during auditory feedback.

During the learning phase, participants in both the TGP tACS and sham groups modified their speech production by reducing their F1 and increasing their F2 values. Contrary to our hypothesis, no significant differences were observed between the groups regarding adaptation to the upwards F1 shift in auditory feedback at the end of the 30 learning phase trials or at the first 15 trials of the after-effect phase. Euclidean distance analysis revealed that the cumulative adjustments in F1 and F2 frequencies, which contributed to the speech production’s approximation to a target vowel in the vowel space, also did not differ between groups.

Examination of the data in the after-effect phase (see Figure 2A), revealed a difference between the groups in the rate of return to baseline F1, which occurs when normal feedback is restored. Specifically, the TGP tACS group showed greater retention of the adapted state and slower return to baseline compared with the sham group. This trend suggests that the administration of TGP tACS during a speech adaptation task might improve the durability of learning. Furthermore, it suggests that theta-gamma activity is a crucial mechanism in reinforcing the persistence of sensorimotor learning in speech.

### No Effect of tACS in the Final 30 Learning and the Initial 15 After-Effect Trials

Our findings revealed that administering TGP tACS over the speech representation in the left M1 did not significantly affect the magnitude of adaptation in F1 and F2 compared with the sham stimulation, in the final 30 learning and the initial 15 after-effect trials. This outcome stands in contrast to previous studies that reported significant differences in F1 adaptation between sham and active groups during similar speech adaptation tasks using tDCS (Lametti et al., 2018; Scott et al., 2020). The adaptation magnitude in the sham group in our study was 28%, which is notably higher than the 10% reported for the sham group in Lametti et al.’s study and more in line with the 29% reported for active stimulation over left M1 in the same study and the 28% change reported for active stimulation by Scott et al. (2020). The anomaly among these studies appears to be the performance of the sham group in the Lametti study, which was unusually low and led to a significant group difference between the active and sham groups. Considering these studies together, it is clear that investigation of larger samples and replication of findings are needed for transcranial electrical stimulation (tES) studies. A further consideration is the inconsistency of behavioural effects. In our case, we propose that the adaptation rate might have approached a learning boundary for this type of sensorimotor learning task, making it challenging to detect the modulatory effects of TGP tACS during the last 30 trials of learning and the first 15 trials of the after-effect phases.

The methodological difference in using TGP tACS instead of tDCS could also have contributed to the different outcomes from the previous studies. Our approach involved the application of tACS at a 75-Hz gamma frequency, synchronised with the peak of a 6-Hz theta envelope, aimed at enhancing speech adaptation effects, as previously reported for improving new motor skill acquisition (Akkad et al., 2021; Rustamov et al., 2022). Nonetheless, given that motor learning comprises multiple different processes with varying complexity (for a review, see Krakauer et al., 2019), the applicability of M1 TGP tACS to speech adaptation as a complex motor skill remains to be determined, despite promising effects observed in simpler tasks such as thumb abduction (Akkad et al., 2021). Using tACS over the cerebellum may offer a more promising target since this can modulate visuomotor adaptation (Figueroa-Taiba et al., 2024). There is also a possibility that the specific modulation of theta and gamma rhythms may not effectively enhance the acquisition of new motor skills within the speech motor cortex, where coordination among hundreds of muscles is required.

Our use of a 2-mA current, oscillating from 1 mA peak anode to 1 mA peak cathode, is lower than the 2-mA tDCS current used in prior studies (Lametti et al., 2018; Scott et al., 2020). This might potentially result in a less potent stimulation for facilitating sensorimotor learning in speech. Another difference between our study and the previous study from Lametti and colleagues, is the placement of the cathode electrode. In the previous study, the cathode was positioned in a landscape configuration on the forehead above the right eye, while in our study, the second electrode was placed over the Pz (to eliminate the possibility of inducing phosphenes from the alternating current). However, our current flow for a direct current simulation suggested that the distribution of current flow successfully targeted the speech articulators (see Figure 1). Overall, we believe it is unlikely that the usage of TGP tACS instead of tDCS or our montage of electrodes accounts for the lack of observed modulatory effects on task performance in our study, especially since our stimulation group exhibited a larger adaptation in F1 compared with the previous two tDCS studies.

It is important to note that replicating the behavioural effects of facilitatory noninvasive brain stimulation in neurotypical populations has proved challenging (Guerra, López-Alonso, et al., 2020). tES may demonstrate optimal efficacy in situations where the cortical region targeted for stimulation exhibits atypical functionality. This has been shown in the context of dyslexia, where the phonological impairments were linked to disrupted low-gamma oscillatory activities within the left auditory cortex (Marchesotti et al., 2020). Selective 30-Hz tACS intervention, significantly enhanced phonological processing and reading precision, with improvements being observable immediately post-intervention. Similarly, application of tDCS improved fluency in people who stutter, further supporting the potential selectivity of tES effectiveness in atypical cortical functionality (Chesters et al., 2018; Moein et al., 2022). The absence of significant findings in our current study, might therefore be attributed to the performance of healthy young adults’ neural systems being near to a ceiling for adaptation, which would limit our sensitivity to detect the expected positive impact of TGP tACS (see Wiltshire & Watkins, 2020, for a similar argument).

In tES studies, effects often become more pronounced after multiple sessions, as the neuroplasticity involved in repetitive training can induce changes over time in a task specific manner (Reis et al., 2009; Saucedo Marquez et al., 2013). For instance, Saucedo Marquez et al. (2013) found that anodal-tDCS applied to the M1 area differently impacts learning and memory, depending on the task. Notably, anodal tDCS over three consecutive days significantly enhanced learning in the sequential finger tapping task, while it only improved retention in the visual isometric pinch force task, in which participants aim to match their pinch grip force to a target level shown on a screen without moving their hand or fingers. Similarly, Reis et al. (2009) observed that repeated tDCS applications over five consecutive days greatly improved skill acquisition in a similar pinch force task, with more substantial improvements by the fifth day. Moreover, Chesters et al. (2017) observed no significant effects following a single session in a within-subject cross-over design involving people who stutter, yet a subsequent 5-day randomised controlled trial showed significant increase in speech fluency (Chesters et al., 2018). This highlights the importance of continuous, daily sessions in enhancing the consolidation of motor skills, suggesting that repetitive tES application can have larger effects on learning outcomes over time. Further research in sensorimotor learning in speech could benefit from this approach, with the application of tES over multiple consecutive days to make the analysis more sensitive to detect group differences.

Another approach would be the use of a within-subject design, as reported in a study that facilitated implicit motor learning using tDCS (Nitsche et al., 2003). In that study, each participant acted as their own control by receiving various stimulation conditions: anodal, cathodal, and sham. This approach would increase statistical power by reducing the impact of between-subject variability. This is particularly relevant because, unlike in visuomotor adaptation studies where nearly every participant adjusts their behaviour after receiving incorrect feedback (Krakauer et al., 2000), a considerable portion of people participating in sensorimotor learning in speech research fail to show any adaptation when experiencing changes in auditory feedback (Rochet-Capellan et al., 2012). While a potential concern in adopting a within-subject design for sensorimotor learning in speech could be the persistence of learning effects over time (Houde & Jordan, 2002), this evidence came from whispered speech, and its applicability to voiced speech remains uncertain. Moreover, recent evidence showed that adaptation for voiced speech exhibits reasonable test–retest reliability (Kapsner-Smith et al., 2024), suggesting that within-participant design may still be feasible.

In summary, even though our study encompassed a larger sample size than previous speech adaptation studies using tDCS and had adequate statistical power to reproduce the original effects, we failed to replicate an effect of this form of brain stimulation in enhancing speech motor learning.

### TGP tACS Enhances Durability of Sensorimotor Learning in Speech

Upon examining Figure 2A, we noticed a difference in F1 values between the groups during the after-effect phase. To assess this difference, we conducted a nondirectional *t* test, which revealed that the mean value for the TGP tACS group was significantly lower than that of the sham group, suggesting sustained learning effects. We believe this broader analysis of all 90 utterances allowed us to better capture the persistence of adaptation and provide a more comprehensive evaluation of sensorimotor learning durability. However, it should be noted that this analysis was exploratory and that removal of an outlier in the sham group reduced the significance of this difference.

We interpret the enhanced durability of sensorimotor learning observed during the after-effect phase to result from neuroplasticity effects induced by gamma stimulation at the peak of theta oscillations. This extended duration of speech motor learning aligns with previous findings that 75-Hz activity (gamma stimulation), particularly when synchronised with theta oscillations, is important for skill acquisition (Nowak et al., 2017). In hippocampal learning, the coupling of specific gamma frequencies, especially the 60–80 Hz range, with the peak of a theta wave associated with memory encoding (Lopes-dos-Santos et al., 2018). The phenomenon of theta-gamma phase-amplitude coupling is recognised not just in the hippocampus but across cortical areas, suggesting its foundational role in cortical computation (Alekseichuk et al., 2016; Fries, 2009; Lisman & Jensen, 2013). Supporting this, Akkad and colleagues (2021) have demonstrated the potential of 75-Hz stimulation, at the peak of 6-Hz oscillations, to enhance motor skill learning. Therefore, our choice of 75-Hz stimulation was influenced by its role in hippocampal learning and its presence in motor cortex gamma activity at theta peaks (Canolty et al., 2006), as well as by positive evidence of its effectiveness in motor skill acquisition (Akkad et al., 2021).

The neurophysiological basis of this finding may stem from GABAergic activity within the motor cortex, which is fundamental to motor plasticity (Clarkson et al., 2010; Stagg et al., 2009, 2011). While reduced gamma-aminobutyric acid (GABA) levels have been associated with enhanced motor learning in some contexts (Stagg et al., 2009, 2011), recent findings suggested higher baseline GABA concentrations in M1 were linked to greater retention of adaptation, though not to learning magnitude (Nettekoven et al., 2021). This distinction between acquisition and retention is relevant to our findings as no TGP tACS effect was measured during the adaptation phase itself; however, our exploratory analysis revealed a prolonged after-effect in the TGP tACS group compared with the sham group. Theta-gamma phase-amplitude coupling could potentially be a key driver of this neuroplasticity (Nowak et al., 2017). Further research employing TGP tACS alongside a neuroimaging method such as magnetic resonance spectroscopy would be beneficial to understand the neurophysiology behind enduring effects of sensorimotor learning in speech.

This trend of prolonged motor learning is consistent with research indicating that M1 plays an important role in maintaining acquired movement patterns once feedback disturbances are removed (Galea et al., 2011; Sale & Kuzovina, 2022; Yamaguchi et al., 2020). For instance, applying anodal tDCS to M1, did not influence the speed or accuracy of visuomotor adaptation but did enhance the durability of the adaptations in the absence of perturbations (Galea et al., 2011). A tACS study that investigated the effects of active 0.75-Hz stimulation on M1 during a ballistic thumb abduction motor training task found similar results (Sale & Kuzovina, 2022). The primary measure of interest was the change in thumb abduction acceleration, assessed immediately after training and again 24 hours later to evaluate short-term and longer term training effects, respectively. Both active and sham stimulation groups showed an immediate increase in thumb acceleration following training; however, only the active tACS group achieved a significant enhancement in thumb acceleration 24 hours post-training, suggesting that 0.75-Hz tACS applied during motor training can improve the retention and possibly the efficiency of motor learning. Another study found that beta-band tACS applied immediately after skill acquisition significantly improved motor skill retention over 1 and 7 days compared with a sham group, suggesting tACS on M1 enhances the consolidation of motor skills (Yamaguchi et al., 2020). Consequently, incorporating a 24-hour post-training assessment in future sensorimotor learning in speech studies could provide valuable insights into the enduring effects of TGP tACS.

We believe that the difference between groups during the after-effect phase is not simply due to increased variability in behavioural responses from TGP tACS, contrary to previous suggestions that noninvasive brain stimulation enhances behavioural variability and thus skill improvement (Teo et al., 2011). Rather, the learning trajectory during the stimulation was consistent across groups until the removal of the F1 perturbation, potentially indicating a direct influence of TGP tACS on the durability of learning (see Figure 2A). Additionally, one might argue that TGP tACS inhibited the unlearning process (also referred as washout) during the after-effect phase, thereby causing group differences. However, this seems unlikely to account for the positive outcomes. If TGP tACS had an inhibitory effect, it should have been evident during the learning phase as well.

### Conclusion

Our study explored the impact of TGP tACS on sensorimotor learning in speech by targeting the speech motor cortex. Contrary to our initial hypothesis and previous tDCS studies, we observed no significant differences between groups in adaptation for both the shifted (F1) and unshifted (F2) formants during the final 30 trials of the learning phase and the initial 15 trials of the after-effect phase. Our findings suggest that the lack of observed modulatory effects on sensorimotor learning is unlikely due to the use of TGP tACS in lieu of tDCS, particularly given that the adaptation in F1 within the sham group in a previous study was 10%, compared with 28% in the sham group of our study. In exploratory analysis, we found reduced mean F1 values in the TGP tACS group throughout the after-effect phase, suggesting an enhanced durability of sensorimotor learning in speech. This finding aligns with previous tACS studies that have shown an increase in the persistence of motor learning. It uniquely highlights the potential significance of theta-gamma coupling in improving the durability of speech motor learning. Nevertheless, these findings are preliminary and highlight the need for further research to confirm their reliability and to further understand the effects of TGP tACS on speech motor learning.

## ACKNOWLEDGMENTS

We thank all the participants for their contributions to this study. We thank Dr. Valentina Mancini, Dr. Camille Lasbareilles, and Qiming Yuan, who assisted with data collection. To facilitate open access, the author has applied a Creative Commons Attribution (CC BY) public copyright license to any author accepted manuscript version resulting from this submission. We are deeply grateful to the Dominic Barker Trust (Registered Charity No. 1063491) for their invaluable support of our research. The views expressed are those of the author(s) and not necessarily those of the NIHR or the Department of Health and Social Care.

## FUNDING INFORMATION

Birtan Demirel, Dominic Barker Trust (https://dx.doi.org/10.13039/100008101). Charlotte J. Stagg, NIHR Oxford Biomedical Research Centre (https://dx.doi.org/10.13039/501100013373), Award ID: NIHR203316. The Wellcome Centre for Integrative Neuroimaging was supported by core funding from the Wellcome Trust (203139/Z/16/Z and 203139/A/16/Z; https://dx.doi.org/10.13039/100010269). Charlotte J. Stagg, Wellcome Trust (https://dx.doi.org/10.13039/100010269), Award ID: 224430/Z/21/Z.

## AUTHOR CONTRIBUTIONS

**Birtan Demirel**: Conceptualization: Equal; Data curation: Lead; Formal analysis: Equal; Investigation: Equal; Methodology: Supporting; Project administration: Lead; Software: Supporting; Validation: Supporting; Visualization: Equal; Writing – original draft: Lead; Writing – review & editing: Equal. **Daniel Lametti**: Formal analysis: Lead; Methodology: Equal; Software: Equal; Supervision: Equal; Validation: Equal; Visualization: Equal; Writing – review & editing: Supporting. **Noa Alony Gilboa**: Data curation: Equal; Formal analysis: Supporting; Project administration: Supporting. **Charlotte J. Stagg**: Conceptualization: Equal; Data curation: Supporting; Formal analysis: Equal; Investigation: Equal; Methodology: Equal; Project administration: Equal; Resources: Equal; Software: Equal; Supervision: Equal; Validation: Equal; Visualization: Equal; Writing – original draft: Equal; Writing – review & editing: Equal. **Kate E. Watkins**: Conceptualization: Lead; Data curation: Supporting; Formal analysis: Equal; Funding acquisition: Lead; Investigation: Equal; Methodology: Equal; Project administration: Equal; Resources: Equal; Supervision: Lead; Validation: Lead; Visualization: Supporting; Writing – original draft: Supporting; Writing – review & editing: Lead.

## DATA AVAILABILITY STATEMENT

The datasets analysed during the current study are available on the Open Science Framework (OSF) at https://osf.io/mt8zx/. This includes the F1 and F2 values for each participant across all phases of the experiment. Additionally, we shared the scripts of the tACS protocol, experimental design and analysis of data.

## TECHNICAL TERMS

Transcranial alternating current stimulation (tACS): A noninvasive brain stimulation method that delivers sinusoidal electrical currents to modulate neural oscillations.
Formant: Frequency bands in speech that characterise vowel sounds and are modified by the vocal tract.
F1: First formant frequency; corresponds to the height of the tongue during vowel production. Changes in F1 affect vowel characteristic, with lower F1 associated with higher tongue positions.
F2: Second formant frequency; corresponds to the posterior or anterior position of the tongue. Higher F2 values are associated with more front vowel articulations.
Theta-gamma coupling: A form of phase-amplitude coupling where gamma oscillations are modulated by the phase of theta rhythms.
Mel: A perceptual pitch scale that reflects how humans hear frequency differences.
Linear predictive coding (LPC): Used in speech processing to estimate formant frequencies.
Euclidean distance: A metric used to measure the distance between two points in formant space (F1–F2).
